# Physiochemical changes mediated by “*Candidatus* Liberibacter asiaticus” in Asian citrus psyllids

**DOI:** 10.1038/s41598-019-52692-7

**Published:** 2019-11-08

**Authors:** Banafsheh Molki, Phuc Thi Ha, Abdelrhman Mohamed, Nabil Killiny, David R. Gang, Anders Omsland, Haluk Beyenal

**Affiliations:** 1 Gene and Linda Voiland School of Chemical Engineering and Bioengineering, Washington State University, Pullman, Washington, USA; 2 Department of Plant Pathology, Citrus Research and Education Center, IFAS, University of Florida, Lake Alfred, Florida USA; 3 Institute of Biological Chemistry, Washington State University, Pullman, Washington, USA; 4 Paul G. Allen School for Global Animal Health, Washington State University, Pullman, Washington, USA

**Keywords:** Biological techniques, Biotechnology

## Abstract

Plant pathogenic bacteria interact with their insect host(s)/vector(s) at the cellular and molecular levels. This interaction may alter the physiology of their insect vector, which may also promote the growth and transmission of the bacterium. Here we studied the effect of “*Candidatus* Liberibacter asiaticus” (“*Ca*. L. asiaticus”) on physiochemical conditions within its insect vector, the Asian citrus psyllid (ACP), and whether these changes were beneficial for the pathogen. The local microenvironments inside ACPs were quantified using microelectrodes. The average hemolymph pH was significantly higher in infected ACPs (8.13 ± 0.21) than in “*Ca*. L. asiaticus”-free ACPs (7.29 ± 0.15). The average hemolymph oxygen tension was higher in “*Ca*. L. asiaticus”-free ACPs than in infected ACPs (67.13% ± 2.11% vs. 35.61% ± 1.26%). Oxygen tension reduction and pH increase were accompanied by “*Ca*. L. asiaticus” infection. Thus, oxygen tension of the hemolymph is an indicator of infection status, with pH affected by the severity of the infection.

## Introduction

A wide variety of plant pathogens are transmitted by insect vectors^1,2^. Among insect vectors, hemipterans, including psyllids, are classified as the most harmful pest groups because of their rapid reproduction and wide variety of host plants^3^. Psyllids, such as the Asian citrus psyllid (ACP) and the potato psyllid, have become the subject of many studies because of their economic impacts. ACPs feed on citrus phloem sap by inserting their stylets into the vascular system of a plant^4,5^. This feeding process by itself is not necessarily significantly damaging to citrus plants. However, it is during this feeding process that the psyllids acquire and transmit “*Candidatus* Liberibacter asiaticus”, the pathogen agent associated with citrus greening disease (also called Huanglongbing, HLB). HLB is considered the world’s most important citrus disease, as it has destroyed millions of acres of citrus groves throughout the United States and other countries^6^. Because ACPs are the carrier of the causative agent of HLB, “*Candidatus* Liberibacter asiaticus,” psyllids play a critical role in the pathology of this disease. Therefore, developing an understanding of how psyllids harbour and transmit “*Ca*. L. asiaticus” is a critical step for efforts to fight this most serious of citrus diseases.

ACPs transmit “*Ca*. L. asiaticus” in a persistent manner, meaning that “*Ca*. L. asiaticus” multiplies and circulates within the hemolymph of the psyllid^1^. Once infected with the bacterium, which replicates within the insect, a psyllid is infectious for life. Clearly, the conditions within the hemolymph are favorable for “*Ca*. L. asiaticus” growth. Indeed, it has been proposed that the psyllid is the primary *host* of “*Ca*. L. asiaticus”^7^ and citrus trees are intermediaries in the transmission of the bacterium from one insect to another (trans-ovarial transmission does not appear to occur at any significant level^8^). The hypothesized mechanism for the transmission of “*Ca*. L. asiaticus” by ACPs is that while uninfected ACPs feed on phloem sap of an infected tree, the bacterium is transferred from the phloem sap to the midgut of the insect. Soon thereafter, the bacterium moves from the midgut to the hemolymph. Once “*Ca*. L. asiaticus” is present in the psyllid hemolymph, it colonizes multiple tissues within the insect (forming biofilms in the process), including the salivary glands. Later, when the infected insect moves to a new citrus plant, the “*Ca*. L. asiaticus” colonies in the salivary glands slough off bacteria, which are injected via the stylet into the phloem of the new plant during feeding^9–12^. Persistent transmission of “*Ca*. L. asiaticus” is an illustration of a strong interaction between “*Ca*. L. asiaticus” and ACP.

Understanding the epidemiology of a plant disease, including the host-vector-pathogen relationships, is crucial to finding a permanent and effective cure^13^. The interactions between the majority of bacterial pathogens and their vectors are poorly understood^3^. However, a few studies have focused on the effect of “*Ca*. L. asiaticus” on the physiology and metabolism of ACPs. Killiny *et al*. observed that in ACPs infected with “*Ca*. L. asiaticus,” the TCA cycle, glycolysis and respiration were induced^14^. In another study, adenosine triphosphate (ATP) levels were found to be significantly higher in infected ACPs than in “*Ca*. L. asiaticus”-free ACPs^1^. In addition, the expression of ATP synthase subunits (involved in ATP production) was elevated and V-ATPase-V1A was reduced (ATP consumption)^1^. Moreover, ATP accumulation was accompanied by high levels of gamma-aminobutyric acid (GABA) in infected samples, which may result from activation of glutamic acid decarboxylase (GAD). It is possible that GAD is activated in infected ACPs via induction of the TCA cycle, since GABA can be converted to succinate and fed into the TCA cycle. Moreover, pyroglutamic acid was reduced in infected ACPs, which may result from the activation of 5-oxoprolinase, an enzyme that converts pyroglutamate to glutamate, and higher activation could be due to high demand for glutamate to produce GABA^14^. These results demonstrate that “*Ca*. L. asiaticus” significantly changes the conditions inside the ACP body, presumably to favor pathogen growth. For example, “*Ca*. L. asiaticus” induces ATP production and reduces its utilization by the ACP, leading to elevated levels of ATP, which might then be available for the bacterium to use via its ATP translocase^1^. Such changes in metabolite levels due to “*Ca*. L. asiaticus” infection can be expected to change the physiochemical conditions (pH and oxygen) inside the ACP body.

In this study, we tested the hypothesis that “*Ca*. L. asiaticus” infection alters pH and oxygen tension inside ACPs. To this end, pH and oxygen microelectrodes were used to measure the local oxygen tension and pH inside the ACP body directly to determine the extent of such changes for two important components within the psyllid body that can affect “*Ca*. L. asiaticus” growth and replication. Such information is valuable for our efforts to develop methods to culture “*Ca*. L. asiaticus.”

## Results and Discussion

In this study, pH and oxygen tension within the abdomen, specifically in the hemolymph, of “*Ca*. L. asiaticus”-infected and -free ACPs were measured, and the average values of these two parameters were calculated for the hemolymph. In addition, the effect of “*Ca*. L. asiaticus” infection on the physiology of ACPs was investigated. Examples of individual radial distributions of pH and oxygen tension are presented to illustrate their variation within the ACP abdomen and explain the method used to calculate the average values for these parameters. The average pH and oxygen tension (100% oxygen tension corresponds to 21% O_2_ in air) in the hemolymph of ACPs are reported in relation to “*Ca*. L. asiaticus” infection status (infected or uninfected) and “*Ca*. L. asiaticus” level (genome equivalents per insect), respectively.

### Radial pH profiles in ACP abdomen

**Figure 1:**
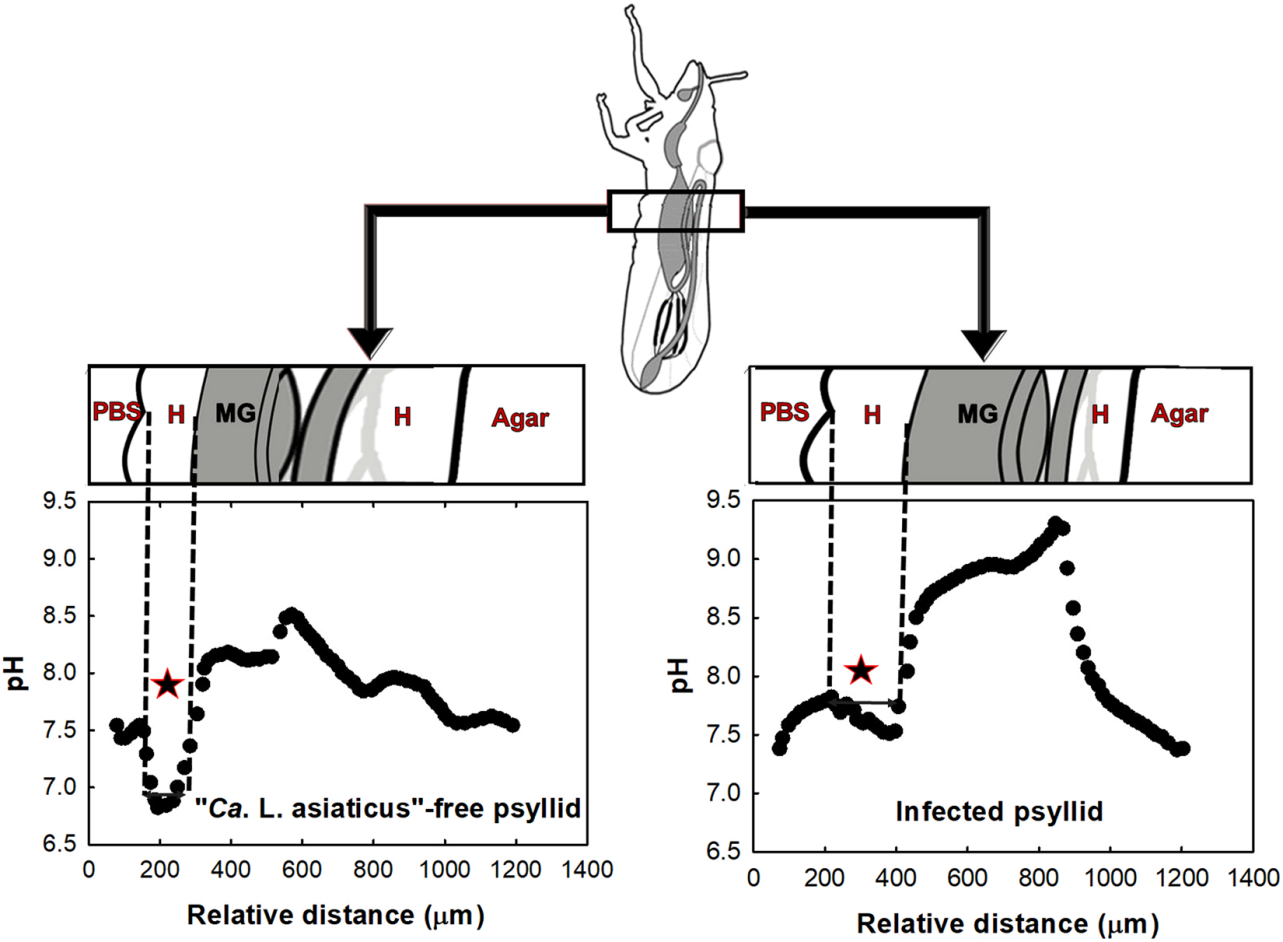
Profiles of pH in “*Ca*. L. asiaticus”-free ACPs and infected ACPs. The region with a star sign is used to calculate the average pH. Hemolymph (H), midgut (MG) and hindgut.

The radial pH distribution in infected and “*Ca*. L. asiaticus”-free ACPs is shown in Fig. 1. Based on the anatomy of ACPs, after penetrating the ACP body, the microelectrode tip first passed through the hemolymph (H) and then into the midgut (MG) and hindgut, and then back into the hemolymph before emerging from the other side of the insect. According to the pH profiles of “*Ca*. L. asiaticus”-free ACPs, the midgut and hindgut are normally alkaline. However, inside the hemolymph, the average pH is close to neutral (7.29 ± 0.15 in H vs. 7.92 ± 0.10 in MG).

It is known that ACPs feeding induces phenolic compound production by citrus leaves^15^, but it is not known to what extent those compounds make it into the phloem, and thus the diet of ACP. However, phenolic compounds in an insect’s diet have damaging effects such as the precipitation of digestive proteins^16^, and it is possible that the midgut alkalinity in insects is an evolutionary adaptation to prevent these damaging effects^16^.

### Radial oxygen profiles of ACP abdomen

**Figure 2:**
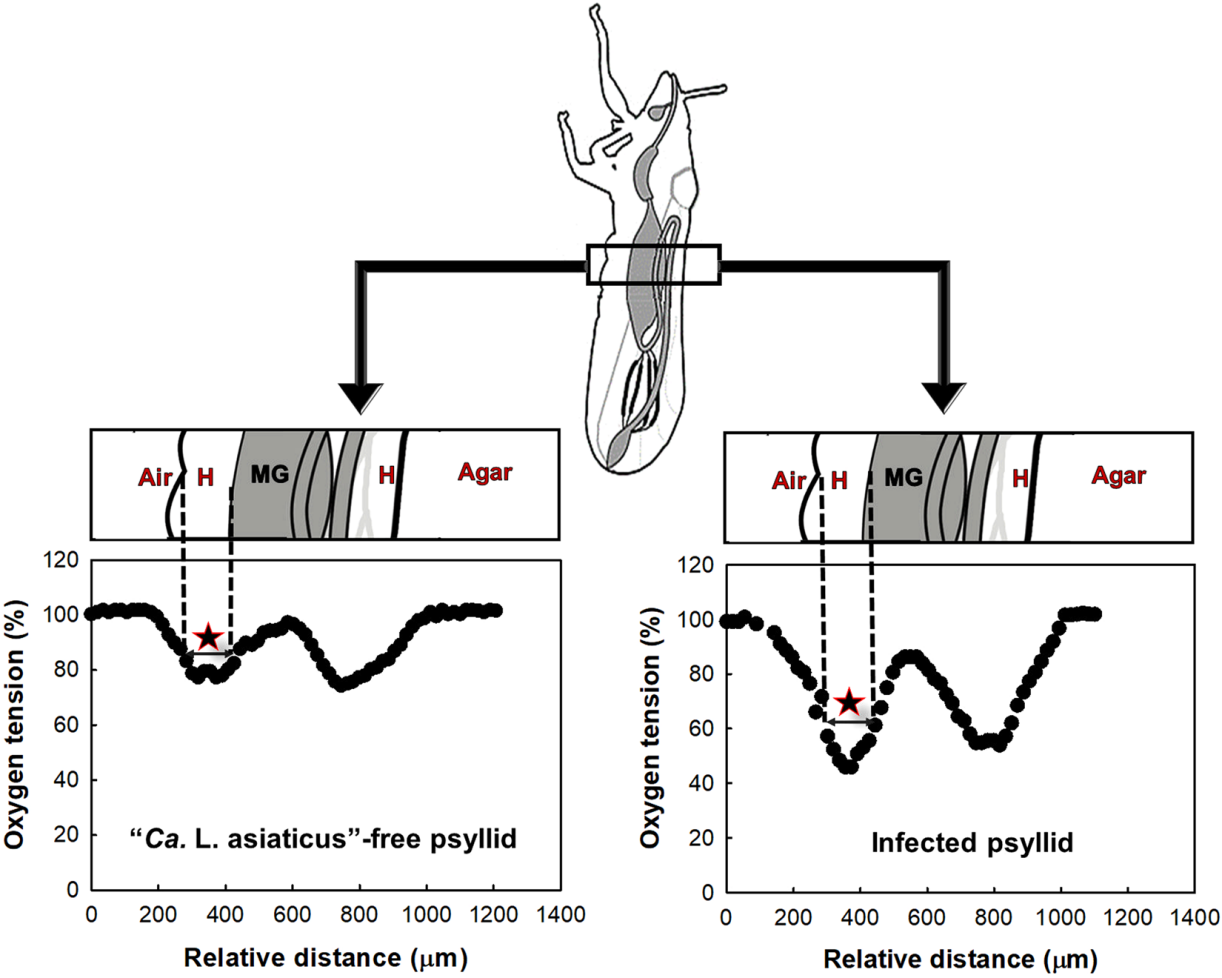
Oxygen tension profiles in “*Ca*. L. asiaticus”-free and infected ACPs. The region with a star sign is used to calculate the average oxygen tension. Hemolymph (H), midgut (MG) and hindgut.

Oxygen tension profiles of “*Ca*. L. asiaticus-free” and infected ACPs are shown in Fig. 2. According to oxygen tension profiles, both hemolymph and midgut lumen of healthy insects contained significant amounts of oxygen. Specifically, the hemolymph oxygen tension was 67.13 ± 2.11% (5.24 ± 0.18 mg·L^−1^) and in the midgut lumen the oxygen tension was 97.91 ± 2.9% (7.76 ± 0.22 mg·L^−1^). Within the hindgut, the oxygen tension was 79.84 ± 1.03% (6.23 ± 0.08 mg·L^−1^). Therefore, the ACP abdomen is aerobic.

The uniform oxygen tension in the hemolymph is expected because of its circulation within the ACP body^17^. The oxygen gradients between different microenvironments in the abdomen are due to dissimilar oxygen consumption or diffusion/transport rates in these microenvironments.

### Effect of “*C*. L. asiaticus” infection level on average pH of hemolymph

**Figure 3:**
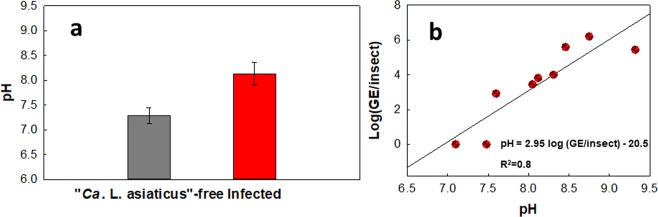
Effects of infection on pH in ACP hemolymph. (**a**) The pH of infected and “*Ca*. L. asiaticus”-free ACPs were significantly different (two-sample t-test: t = 1.75, P = 0.0041). (**b**) There was a significant positive relationship between pH and infection level (pH versus log(GE/insect)). Slope = 2.95, SE = 0.57; linear regression: t = −5.2, P = 0.0012. “*Ca*. L. asiaticus” genomic equivalents (GE) per insect was calculated based on the standard curve, given that each “*Ca*. L. asiaticus” has three identical copies of the 16 S rRNA gene in its genome^30^.

The effect of “*Ca*. L. asiaticus” infection on hemolymph pH in ACPs is shown in Fig. 3. The average hemolymph pH in “*Ca*. L. asiaticus”-free ACPs was 7.29 ± 0.15, and in infected ACPs it was 8.13 ± 0.21. This clearly demonstrates that there was a statistically significant difference between “*Ca*. L. asiaticus”-free and infected average hemolymph pH (two-tailed Student’s t-test, t = 1.75, P < 0.0001). In turn, ACPs with higher infection levels had higher average hemolymph pH (slope = 2.95, SE = 1.092).

**Figure 4:**
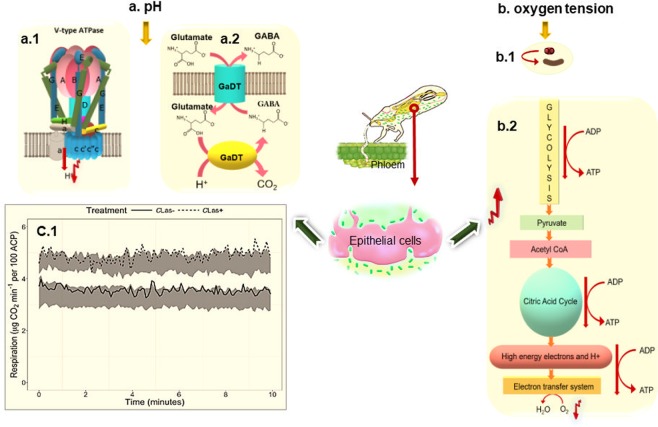
The proposed mechanism for **(a**) increased pH and (**b**) decreased oxygen levels in ACP upon infection by “*Ca*. L. asiaticus”: (a.1) downregulation of ATPase in ACP, (a.2) upregulation of GAD reaction and (c.1) elevated respiration rate^14^. (b.1) Oxygen consumption by *Ca*. L. asiaticus and (b.2) elevated respiration in ACP. Figure c.1 is reproduced from a Killiny *et al*. publication titled “A plant pathogenic bacterium exploits the tricarboxylic acid cycle metabolic pathway of its insect vector”(Copyright © 2018 The Author(s). Published by Informa UK Limited, trading as Taylor & Francis Group. This is an Open Access article distributed under the terms of the Creative Commons Attribution License (http://creativecommons.org/licenses/by/4.0/), which permits unrestricted use, distribution, and reproduction in any medium, provided the original work is properly cited).

It is not completely clear how “*Ca*. L. asiaticus” affects pH within psyllids. Because respiration is affected by the presence of the bacterium (both glycolysis and ATP synthesis appear to be elevated in infected insects^1^), it is possible that organic acids, such as the concentration of TCA cycle intermediates, are affected as well. More extensive metabolite profiling experiments need to be performed to answer this question, particularly experiments that can identify just where within the psyllid body specific metabolic processes are most significantly affected. However, there are at least three known potential mechanisms that could increase pH in ACPs. The first mechanism is the downregulation of the V-ATPase-V1A level. V-ATPase-V1A is an ATP-dependent proton pump localized to a variety of cellular membranes of eukaryotic cells including the outer membrane of mitochondria and the plasma membrane. V-ATPase-V1A pumps use ATP hydrolysis to acidify the environment^18,19^. Therefore, their reduced level can increase the pH in intracellular and extracellular fluid. Second, the higher activation of the GAD enzyme in infected ACPs increases pH. The GAD reaction is proton-consuming and can act as a sink for excess protons and thus contribute to pH increase. Third, a higher respiration rate increases pH and decreases CO_2_ concentration in the hemolymph. The proposed mechanisms are shown in Fig. 4(a).

### Effect of “*Ca*. L. asiaticus” infection on oxygen tension profile in ACP hemolymph

**Figure 5:**
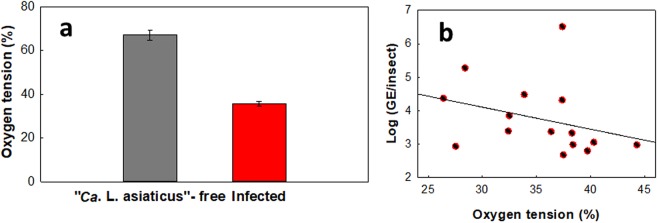
Effect of infection on oxygen tension in ACP hemolymph (%). (**a**) Levels of oxygen tension measured in infected and uninfected ACPs. (**b**) Oxygen tension (%) versus log(GE/insect) in infected ACPs(two-tailed Student’s t-test, t = −11.99, P < 0.0001). No linear regression was observed between oxygen tension and GE/insect (slope = −0.066, SE = 0.055; linear regression: t = −1.19, P = 0.255, R^2^ = 0.09). “*Ca*. L. asiaticus” genomic equivalents (GE) per insect was calculated based on the standard curve, given that each “*Ca*. L. asiaticus” has three identical copies of the 16S rRNA gene in its genome^30^.

The average hemolymph oxygen tensions in “*Ca*. L. asiaticus”-infected and -free ACPs, as shown in Fig. 5, were 35.61% ± 1.26% and 67.13% ± 2.11%, respectively, a difference that was found to be statistically significant (two-tailed Student’s t-test, t = −11.99, P < 0.0001). No linear relationship between oxygen tension and log(GE/insect) was observed.

Oxygen tension reduction in the ACP body may result from two different mechanisms. By one mechanism, oxygen is consumed by “*Ca*. L. asiaticus.” In a previous study, it was shown that Japanese beetles infected with *Bacillus popilliae* (*B*. *popilliae*) had a 15% lower oxygen level than uninfected beetles^20^. It was hypothesized that the oxygen level reduction is due to oxygen consumption by *B*. *popilliae*. It is possible that, similar to Japanese beetles, in infected ACPs, oxygen reduction results from oxygen consumption by “*Ca*. L. asiaticus,” suggesting that the bacterium requires oxygen for growth. By the other mechanism, oxygen tension is reduced via elevated respiration by the insect. In a previous study, it was observed that in infected ACPs, the respiration rate was increased by 42%, which was hypothesized to be due to induction of the TCA cycle^14^. A proposed mechanism for this is outlined in Fig. 4(b). In addition, the oxygen tension reduction could be the reason that GABA accumulates inside the psyllid body. Stress from oxygen deficiency influences the accumulation of GABA^21^. In other words, GAD enzymes become more activated in a reduced oxygen tension condition and, since the GAD reaction is proton-consuming, pH increases.

### Determination of required pH and oxygen tension to culture “*Ca*. L. asiaticus” *in vitro*

A brief discussion of potential metabolic pathways present in “*Ca*. L. asiaticus” accompanied the initial report of the metagenomics sequencing-derived predicted genome of this important pathogen^22^. The authors suggested that the “*Ca*. L. asiaticus” had limited capacity for anaerobic respiration because it putatively contained no homologs for terminal stages in oxidative phosphorylation. However, the two enzymes that were listed as evidence for this (as key components of this well-known and important biochemical pathway) were polyphosphate kinase and inorganic diphosphatase, enzymes that are not actually involved in oxidative phosphorylation but instead are involved in phosphate homeostasis within the cell. Some homologs for potential terminal oxidases or electron transport chain intermediates, the cytochrome bc1 complex, cbb3-type cytochrome c oxidase, and the cytochrome bd complex were reported as missing from the genome sequence^22^. However, all four known cytochrome O ubiquinol oxidases (which can be used in non-canonical oxidative phosphorylation) were found in the genome sequence^22^. In another study^23^ five copies of *nrdB*, encoding the β-subunit of ribonucleotide reductase (RNR), were reported to be encoded by the “*Ca*. L. asiaticus” genome sequence^23^. The *nrdB* gene encodes a key enzyme for converting ribonucleotides to deoxyribonucleotides, the precursors of DNA synthesis and repair. Based on *nrdB* classification, “*Ca*. L. asiaticus” *nrdB* is exclusively oxygen-dependent, which suggests that “*Ca*. L. asiaticus” has an aerobic lifestyle^23^. Moreover, in the previous report by Parker *et al*.,^24^ the authors successfully prolonged “*Ca*. L. asiaticus” viability by supplementing the growth medium with citrus juice under aerobic conditions^24^. Therefore, it is likely that “*Ca*. L. asiaticus” is an aerobe.

One of the most reliable methods to determine the optimum pH for “*Ca*. L. asiaticus” growth is to measure pH in “*Ca*. L. asiaticus” vectors and hosts. In a previous study^25^, the pH of the phloem sap of citrus trees was reported to be 6.04 ± 0.16, which is lower than the pH observed for the psyllid body in this study. This could be one of the reasons that “*Ca*. L. asiaticus” multiplies in microenvironments with lower pH (hemolymph) within the psyllid. In the study by Parker *et al*.^24^ the pH of the culture medium was around 3, much lower than that observed in the ACP abdomen. Low pH may have prevented “*Ca*. L. asiaticus” replication under the conditions tested by Parker *et al*.^24^. In future attempts to culture “*Ca*. L. asiaticus,” it could be advantageous to adjust the pH of the culture medium to a more neutral range. Thus, the results of this study provide key insights into the natural growth environment of “*Ca*. L. asiaticus” that will be helpful in guiding future attempts at its culture.

### Midgut average pH and oxygen tension

**Figure 6:**
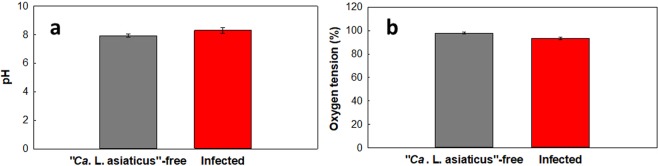
Effects of “*Ca*. L. asiaticus” infection on (**a**) pH and (**b**) oxygen tension in ACP midgut. There are no significant differences in pH (two-tailed Student’s t-test: t = 0.108, P = 0.915) or oxygen tension (two-tailed Student’s t-test: t = −0.758, *P* = 0.455) between “*Ca*. L. asiaticus”-free and infected ACPs.

We also calculated the average pH and oxygen tension of infected and “*Ca*. L. asiaticus”-free ACP midgut. The pH of the midgut of “*Ca*. L. asiaticus”-free ACPs (mean = 7.93, SE = 0.37) was not significantly different from that of the midgut of infected ACPs (mean = 8.31, SE = 0.55; two-tailed Student’s t-test: t = 0.108, P = 0.915) (Fig. 6(a)). The levels of oxygen tension in the midgut of “*Ca*. L. asiaticus”-free ACPs (mean = 97.91%, SE = 2.90) were also not significantly different from those in the midgut of infected ACPs (mean = 93.46% SE = 4.62; two-tailed Student’s t-test: t = −0.758, *P* = 0.455) (Fig. 6(b)). It has been shown that the midgut pH can be affected by feeding conditions^26^. Since the metabolites in infected versus “*Ca*. L. asiaticus”-free citrus tree phloem sap are different^27^, the midgut pH may change because of feeding on infected trees. Therefore, it is not clear whether “*Ca*. L. asiaticus” infection can affect the pH of the midgut. To the best of our knowledge, there is no information available on the effect of psyllid feeding on midgut oxygen tension.

### Practical implications

In this study, we found that the average oxygen tension in the hemolymph of infected ACPs was significantly lower than that of “*Ca*. L. asiaticus”-free ACPs (35.61 ± 1.26% vs. 67.13 ± 2.11%). Therefore, measurement of the oxygen tension in the hemolymph of ACPs can potentially predict the infection status of the ACPs. In addition, there is a statistically significant linear relationship between pH and infection level (genome equivalents per insect). Equation 1 shows the relationship between pH and infection level in ACPs.

1 $${\rm{pH}}={\rm{2.95}}\,\log ({\rm{GE}}/{\rm{insect}})-{\rm{20.5}}$$

In this equation, GE is the genome equivalents of “*Ca*. L. asiaticus” and pH is the average pH of the hemolymph. Based on this equation, it may be possible to predict the GE of “*Ca*. L. asiaticus” in infected ACPs from the average hemolymph pH of the ACPs. However, we should note that these correlations may be different for ACPs of different ages, physiological conditions and symbiont populations.

## Methods

### Psyllids

The ACP colony was originally established using field populations from Polk County, Florida. ACPs in the colony were reared continuously on curry leaf, *Bergera koenigii*, placed into plexiglas chambers and kept under a light:dark 16:8 h photoperiod at 27 ± 2 °C and 60 ± 5% relative humidity. ACPs used for experiments were transferred to uninfected or “*Ca*. L. asiaticus”- infected Valencia sweet orange (*C*. *sinensis* ‘Valencia’) in two separate growth rooms under a light:dark 14:10 h photoperiod at 27–28 °C and 60–65% relative humidity. Both colonies, as well as the citrus plants, were routinely monitored using PCR to confirm infection with “*Ca*. L. asiaticus.” Adult “*Ca*. L. asiaticus”- infected psyllids were sampled without categorizing by age or sex when the “*Ca*. L. asiaticus” infection percentage rose above 50%.

### Construction of pH microelectrodes

**Figure 7:**
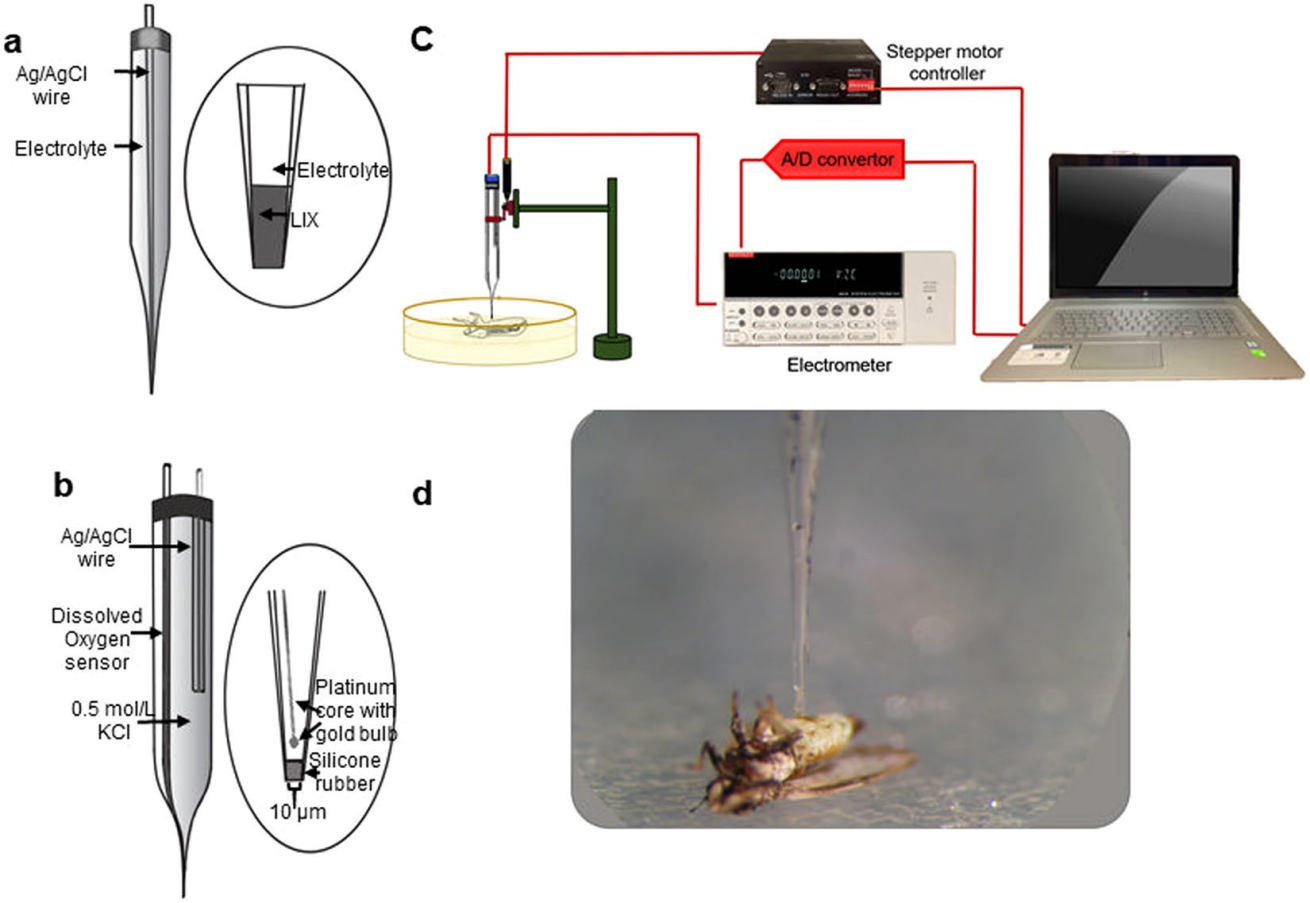
Schematics of (**a**) pH and (**b**) oxygen microelectrodes^28^. The pH microelectrode requires a reference electrode (not shown), which can be located near the microelectrode. (**c**) Measurement setup used to measure oxygen and pH profiles in ACPs. (**d**) ACP on agar plate before start of measurement. The wings of the ACP were fixed using small drops of agar. ACPs were laid on their backs, and the microelectrode penetrated their abdomens. The figure is not drawn to scale.

A pH microelectrode is a potentiometric microelectrode that measures pH based on the potential difference across a liquid ion exchange (LIX) membrane located on the tip of the microelectrode. The schematics of a pH microelectrode are shown in Fig. 7(a). The construction details are given in our previous publication^28^. The microelectrode potential was measured against a separate Ag/AgCl reference electrode in various pH buffers (4, 7 and 10) to generate a calibration curve. The pH microelectrodes had tip diameters of less than 20 µm and response times of less than 3 seconds.

### Construction of oxygen microelectrodes

The oxygen microelectrode is a needle-type microelectrode (Fig. 7(b)). It detects the oxygen concentration at the tip in both liquid and gas phases. The microelectrode uses a gold cathode and an Ag/AgCl reference/counter electrode. Oxygen diffuses through a silicone rubber membrane and then is reduced at the gold cathode. The reduction current is proportional to the oxygen concentration in the vicinity of the microelectrode tip. The construction process is detailed in our previous publication^28^. The oxygen microelectrodes we used had tip diameters of less than 20 µm and response times of less than 3 seconds. The gold cathode was polarized at −0.8 V against Ag/AgCl to operate the oxygen microelectrode. The microelectrodes were calibrated using two-point calibration: in Na_2_SO_3_ solution (zero oxygen concentration) and in the air.

### Measurement of pH and oxygen profiles

The setup and instruments that were used for microelectrode measurements are shown in Fig. 7. Oxygen profile measurements were performed on 16 infected and 14 “Ca. L. asiaticus”-free ACP individuals.

Each ACP was used for a single measurement. For each measurement, a live ACP was fixed on its back on a 2.5% agar layer (Fig. 7(c)). A measurement was completed in less than 5 min for each ACP. The oxygen tension measurement started a little above the abdomen and continued until the microelectrode emerged from the other side of the abdomen. The measurement stopped when the tip of the microelectrode reached the agar layer below the insect. A mercury-step stepper motor (PI M-230.10 S) was used to control the movement of the microelectrode. This stepper motor was connected to a computer, and the relative distance of the microelectrode tip was determined with custom-made Microprofiler© software developed by our research group. Data were recorded in the computer using a data logger (Measurement Computing© USB-1608FS).

The pH profile measurements were performed in the same manner as the oxygen profile measurements except that we added a few drops of phosphate-buffered saline with pH = 7.5 (PBS, 100–300 µL) to cover the insect’s surface. The microreference electrode was inserted into the PBS, and then the procedures for measuring pH with oxygen microelectrodes were followed. The potential between the reference electrode and the pH microelectrode was measured, and then the potential values were converted to pH values using the calibration curve for this microelectrode. For pH measurements, 9 infected and 13 “*Ca*. L. asiaticus” -free ACP individuals were tested.

### DNA extraction and quantitative PCR for “*Ca*. L. asiaticus” detection

A heating method described previously^29^, but with several modifications, was used to prepare ACP DNA extracts. Individual ACPs were washed with washing buffer, and then each was put in a mortar containing 300 µL of extraction buffer (10 mM Tris and 1 mM EDTA, pH 8.0) and crushed completely using a pestle. The crude ACP extract solution was transferred to a Lysing Matrix E tube (MP Biomedicals) and vortexed for 10 min, followed by centrifugation (16100 × g for 90 min), with the aqueous portion of each sample being transferred to a PCR tube. The samples were then heated at 80 °C for 7 min and then at 95 °C for 3 min, followed by centrifugation at 8000 × *g* for 2 min. The aqueous portion of each sample was used as a DNA template in a final PCR reaction volume of 200 µl. Quantitative PCR using the following primer pair was performed to quantify “*Ca*. L. asiaticus” DNA in each sample: 5′-GGT TTT TAC CTA GAT GTT GGG TAC T-3′ and 5′-CTT CGC AAC CCA TTG TAA CC-3′. Both of the primer sequences are conserved in “*Ca*. L. asiaticus” and were designed to amplify the Las-specific DNA fragment (140 bp) from its 16S rRNA gene. The reaction mixture components and target concentrations were a qPCR master mix (Power SYBR Green PCR Master Mix, Applied Biosystems, USA) with a final concentration of 1×, forward primer and reverse primers with a final concentration of 0.2 µM each. The DNA volume for each reaction mixture was 2 µl. The cycling parameters used to run the quantitative PCR were 94 °C for 2 min, followed by 40 cycles of 94 °C (denaturation) for 15 s and 60 °C (annealing, extension, and red fluorescence) for 1 min.

The specific 16S-rRNA amplicon (1160 bp) for “*Ca*. L. asiaticus” was obtained using PCR with high-fidelity DNA polymerase and the conventional primers (Ol1, Ol2c)^30^, purified and used as the molecular standard for qPCR. A 10-fold dilution series was prepared to create the standard curve. The genomic equivalents (GE) per insect was calculated based on the standard curve, given that each “*Ca*. L. asiaticus” has three identical copies of the 16S rRNA gene in its genome^30^.

### Data analysis

Two-sample *t*-tests were used to determine whether there was a statistical difference in pH or oxygen tension between “*Ca*. L. asiaticus”-free and infected ACPs. We used linear regression to explore the relationships between pH and genome equivalents/insect (GE/insect) values and between oxygen tension and genome equivalents/insect (GE/insect) values. All statistics were computed using Excel 2013.

### Location of the microelectrode tip within the ACP body

Based on a sharp change in profile, the microelectrode tip is exposed to new conditions, which in turn indicates that it has entered a new environment. Based on the ACP’s anatomy, the microelectrode tip passes through three different environments. Therefore, by comparing the profiles with ACP anatomy, it is possible to predict the location of the microelectrode tip.

When the microelectrode tip penetrates into the midgut, a hole is formed on the wall of the gut. This is expected to cause insignificant leakage of midgut lumen into the hemolymph, and because of the circulation of midgut lumen in the ACP body, this phenomenon may increase the pH of the hemolymph. Thus, to calculate the average pH of the hemolymph, the pH of the region that is highlighted with a star in Fig. 1 was used to make sure that gut lumen had not changed the pH of the hemolymph.

## Data Availability

The authors confirm that the data supporting the findings of this study are available within the article. Raw data supporting the findings of this study are available from the corresponding author (HB) on request.
